# The viability of an ecologically valid chronic sleep restriction and circadian timing protocol: An examination of sample attrition, compliance, and effectiveness at impacting sleepiness and mood

**DOI:** 10.1371/journal.pone.0174367

**Published:** 2017-03-20

**Authors:** David L. Dickinson, Sean P. A. Drummond, Todd McElroy

**Affiliations:** 1 Department of Economics & Center for Economic Research and Policy Analysis, Appalachian State University, Boone, North Carolina, United States of America; 2 IZA (Institute for the Study of Labor), Bonn, Germany; 3 ESI (Economic Science Institute), Chapman University, Orange, California, United States of America; 4 Monash Institute of Cognitive and Clinical Neuroscience, School of Psychological Sciences, Monash University, Melbourne, Victoria, Australia; 5 Department of Psychology, Florida Gulf Coast University, Ft. Myers, Florida, United States of America; University of Pennsylvania, UNITED STATES

## Abstract

Chronic sleep restriction (SR) increases sleepiness, negatively impacts mood, and impairs a variety of cognitive performance measures. The vast majority of work establishing these effects are tightly controlled in-lab experimental studies. Examining commonly-experienced levels of SR in naturalistic settings is more difficult and generally involves observational methods, rather than active manipulations of sleep. The same is true for analyzing behavioral and cognitive outcomes at circadian unfavorable times. The current study tested the ability of an at-home protocol to manipulate sleep schedules (i.e., impose SR), as well as create a mismatch between a subject’s circadian preference and time of testing. Viability of the protocol was assessed via completion, compliance with the SR, and success at manipulating sleepiness and mood. An online survey was completed by 3630 individuals to assess initial eligibility, 256 agreed via email response to participate in the 3-week study, 221 showed for the initial in-person session, and 184 completed the protocol (175 with complete data). The protocol consisted of 1 week at-home SR (5-6 hours in bed/night), 1 week wash-out, and 1 week well-rested (WR: 8-9 hours in bed/night). Sleep was monitored with actigraphy, diary, and call-ins. Risk management strategies were implemented for subject safety. At the end of each experimental week, subjects reported sleepiness and mood ratings. Protocol completion was 83%, with lower depression scores, higher anxiety scores, and morning session assignment predicting completion. Compliance with the sleep schedule was also very good. Subjects spent approximately 2 hours less time in bed/night and obtained an average of 1.5 hours less nightly sleep during SR, relative to WR, with 82% of subjects obtaining at least 60 minutes less average nightly sleep. Sleepiness and mood were impacted as expected by SR. These findings show the viability of studying experimental chronic sleep restriction outside the laboratory, assuming appropriate safety precautions are taken, thus allowing investigators to significantly increase ecological validity over strictly controlled in-lab studies.

## Introduction

There is a large volume of in-laboratory experimental data showing the negative impact of chronic sleep restriction (SR) and/or unfavorable circadian timing on alertness, mood, and performance [1,2,3,4,5]. These multi-day studies have provided foundational data defining adverse consequences, identifying relevant mechanisms, and setting the stage for more applied research. However, intensive multi-day lab studies require significant financial and personnel resources, involve extensive levels of control not experienced in everyday life, and can only recruit individuals capable of living in the lab for multiple consecutive days. These factors limit the feasibility and sample size of such studies, as well as the generalizability of their findings. As a result, the extent to which findings apply to the types of sleep restriction and circadian timing effects experienced in everyday life remains unclear.

Thus, there is the need for a protocol design that allows investigators to study large groups of individuals in a naturalistic environment. Such a protocol should allow subjects to go about their daily lives while also allowing for investigator-determined levels of SR and timing of assessments. This would provide a balance between tightly controlled laboratory studies and completely uncontrolled observational studies. One model approaching these goals is the study of shift workers outside the lab. These studies have shown both SR and circadian timing effects on various outcomes [6,7,8,9]. While valuable, that model does not address non-shift workers who may nonetheless restrict their sleep and attempt to perform during the extreme times of the “normal” day.

Some methodologies have included either SR or circadian misalignment in naturalistic settings other than shift work. The SR protocols have largely involved children or adolescents [10,11,12,13]. Such protocols necessarily require parental involvement, and the complementary efforts of the minor’s parents likely increase compliance in a way that cannot be expected in an adult study. To our knowledge, only one prior study has assessed the validity of an in-home SR protocol in young adults [14], while two others included at-home SR as part of larger designs [15,16]. The first study [14] utilized a relatively small sample of 34 university undergraduates, and the subjects underwent only one well-rested (WR) and one SR evening each. Generally, SR protocols are interested in longer periods of SR and, when SR is verified by actigraphy, a minimum of 5 nights of data is recommended [17].

The other two noteworthy studies each pursued at-home SR with different objectives. In one [15], a small sample (21 young adults) was studied using an at-home full-week sleep manipulation in conjunction with a 36-hour total sleep deprivation (TSD) protocol. Subjects each completed three replications of the TSD protocol with either an at-home extended sleep week (12 hrs/night in bed) or an at-home SR week (6 hr/night in bed). In that study, the authors argued against such at-home SR protocols for safety reasons. However, compared to ours their study appears to have more stringently promoted at-home SR. Their subjects averaged only 4.6 ± .8 hrs/night sleep, while ours averaged 5.6 ± .6 hrs/night during at-home SR. In another study [16], at-home SR involved only a single night of 5 hrs/time-in-bed prior to more restrictive in-lab SR. Compared to our protocol, that study imposed more severe at-home SR (though just for a single night), more fully screened subject health prior to enrollment, but also prohibited subjects from consuming caffeine during the at-home SR.

The nightly sleep levels we report are more similar to self-selected sleep levels reported in observational studies [18,19] and recent survey data [20] for many young adults, and our allowance of caffeine intake both enhances the external validity of our protocol and represents a risk management feature of our design. It is clear that a careful weighing of risks and benefits is important in designing an at-home sleep study. A protocol that allows one to examine the consequences of these more commonly experienced sleep levels under naturalistic conditions, and attribute causation more clearly than with observational data, is important and this was our primary design objective. We more fully review in the Discussion section the measures we implemented to manage the inherent risk in a protocol designed to induce sleepiness outside of a sleep-lab setting.

Previous circadian protocols have randomly matched or mismatched subjects to a more- or less-preferred time of day, relative to the subject’s self-reported diurnal preference [21,22]. However, these earlier papers gathered morningness-eveningness scores only after recruitment, and then utilized a median split to define morning-type (MT) versus evening-types (ET). That methodology is problematic in the college student samples they utilized, because such samples will likely yield no more than 10% MTs given the known characteristics of the diurnal preference distribution of young adults [23]. Alternatively, more recent circadian protocols predetermined MT and ET status prior to recruitment so that roughly equal numbers of each are randomly matched and mismatched to session times [24,19]. These circadian mismatch protocols significantly increased self-reported sleepiness and significantly altered behavioral outcomes on high-level decision tasks.

The aim of the current study was to design a cost-effective protocol where investigators can study relatively large samples in naturalistic settings while still allowing control over sleep duration, assessment timing, and the circadian match/mismatch associated with time of testing. Given the known relationship between SR and a wide range of adverse outcomes (increased accident risk [25,26,27], sleepiness, reduced concentration, slower reaction times, adverse mood states [1,4,15,28]), we carefully weighed the costs and benefits in our protocol and later discuss our risk management strategies in more detail in the Methods and Discussion sections. While we hypothesized that both the SR and circadian mismatch would increase subjective sleepiness, the analysis was more exploratory regarding the determinants of compliance. Overall, our aims were to determine: a) rates and predictors of protocol completion; b) level of compliance with prescribed SR; and c) the effects of the resultant SR and circadian match/mismatch on subjective sleepiness and mood ratings. If successful, the protocol would provide a design readily applied in a multitude of natural environments, and in such a design it would be easy to include cognitive or other performance testing measures at the end of each treatment condition.

## Methods

### Subject recruitment and screening

We first administered a large-scale online survey meant to provide information on subject sleep habits (see S1 Appendix for online survey instruments). The survey link was included within an announcement emailed to randomly drawn subsamples of a college campus student community (e.g., 3000 drawn from Juniors and Seniors, of which a few hundred would typically complete the survey during each survey wave). Announcements were also emailed to faculty and staff, and online ads were posted to the local non-University community. As might be expected, the majority of responses were from students. The initial online survey was approved by the Institutional Review Board (IRB) in the Office of Research Protection at Appalachian State University. The survey started with a required consent page before one could continue the survey. The IRB approved this written consent procedure for the online survey. In addition to asking for basic demographic information, the survey administered a set of validated screener questions for anxiety (the 7-item Generalized Anxiety Disorder instrument, GAD-7 [29]) and the 2-item Patient Health Questionnaire (PHQ-2) for depression risk assessment [30]). The following individuals were excluded from our study: those younger than 18 or older than 39 years of age, those with a self-reported insomnia or a diagnosed sleep disorder, or those who scored at risk of major depressive (PHQ-2 score > 2) or anxiety disorder (GAD-7 score > 9) given the correlation between these conditions and sleep disturbance. Beyond the PHQ-2, the GAD-7, and self-reported sleep disorders, we did not screen for other existing medical conditions, although other researchers may choose to implement additional screening criteria. For example, in related studies only healthy normal sleepers are recruited, which would exclude those with substance addictions [14,15,16]. In our efforts to recruit a maximally representative sample and maintain external validity, we did not screen for substance use disorders. Importantly, within the online survey we also included a validated measure of diurnal preference: the reduced form of the morningness-eveningness questionnaire (rMEQ, [31]), which is a reduced scale version of the original Horne and Ӧstberg scale [32]. The rMEQ classifies individuals on a scale of 4-25, with morning-types having rMEQ score from 18-25 and evening-types having rMEQ score from 4-11.

We included additional questions within the survey to gather self-report sleep data and Epworth Sleepiness scores (ESS: trait-level daytime sleepiness [33]). In terms of self-report sleep data, subjects were asked to report their average amount of nightly sleep during the full week preceding the survey, as well as the amount of sleep the night immediately preceding the survey. Subjects were also asked to self-report the amount of nightly sleep considered personally optimal, in terms of alertness and performance (see S1 Data for all raw data).

Two sets of subjects were recruited: 1) individuals who were either morning-types (MT) or evening-types (ET), who were then assigned to a treatment condition schedule of one SR and one WR week; and 2) individuals in the middle range of rMEQ scores who were labeled as “indeterminate” or intermediate-types (IT). These IT subjects were assigned two weeks of WR, rather than one SR and one WR week, and served as control subjects.

### Determination of circadian match/mismatch testing time

Based on rMEQ scores from the online survey, we first identified MT and ET subjects and randomly assigned half of each to morning (7:30 a.m.) or evening (10:00 p.m.) test sessions. This resulted in approximately half of our treatment sample being circadian matched and half mismatched for the test sessions. Control subjects, who did not display strong diurnal preference, were tested at 11:00 a.m. so that circadian mismatch was not a concern for them. Table 1 shows the distribution of the circadian manipulation in our sample of treatment subjects. Note that, due to the rarity of true MT subjects [23], we extended our rMEQ morning-type cutoff to include rMEQ scores of 16 and 17. To compensate, we shifted the evening-type cutoff an equal amount and only recruited ET subjects with rMEQ scores from 4-9. In this way, our sample is still drawn from non-central rMEQ scores and preserves the same separation between MT and ET subjects as if using originally suggested score cutoffs. In the end, we achieved good balance between circadian mismatched/matched subjects, though our sample included more female than male subjects (this is true in the viable subject database as well): Mismatched n = 44F,32M (age = 21.21 ± 3.87 years old); Matched n = 48F,25M (age = 21.95 ± 4.71 years old)

**Table 1:** Final sample size per design cell (treatment subjects).

	Morning Session	Evening Session
**Morning-type**	**34 (30)**	**38 (28)**
**Evening-type**	**38 (29)**	**39 (32)**

**Notes:** Circadian mismatches cells shaded. Sample Size n = 149 subjects (Mismatched obs = 76, Matched obs = 73). Compliant & sleep data intact, n = 119, shown in parenthesis.

### Sleep restriction

Whether circadian matched or mismatched, all treatment subjects were prescribed one SR and one well-rested (WR) week during the 3-week protocol, with a week of ad lib sleep as a wash-out in between the two experimental treatment weeks. During SR, subjects were asked to spend 5-6 hrs/night in bed attempting to sleep, whereas during WR subjects were asked to spend 8-9 hrs/night in bed attempting to sleep. Naps were discouraged but did not disqualify a subject. However, naps would contribute to one’s average daily sleep quantity and increase the likelihood of being deemed noncompliant (see Discussion for additional information on naps in our sample). We highlight in the Discussion section the risk management measures we put in place due to the sleepiness anticipated from the circadian mismatch and SR components of the protocol. Compliance with the prescribed sleep levels was measured with research-grade actigraphy (Actiwatch Spectrum devices, Philips Respironics), complemented with data from a daily sleep diaries and phone call-ins every evening and morning from each subject to verify bed and wake times (although one might now prefer using email or texts). Actigraphy has been established as a reliable and valid method for the naturalistic study of sleep levels in healthy normal sleeping adults (see [34] for a survey discussing validity and limitations of actigraphy). The order of the experimental treatment weeks was randomly assigned to each cohort of subjects (so everyone in a given cohort was always in the same condition) and counterbalanced across cohorts.

### Test sessions

**Fig 1:**
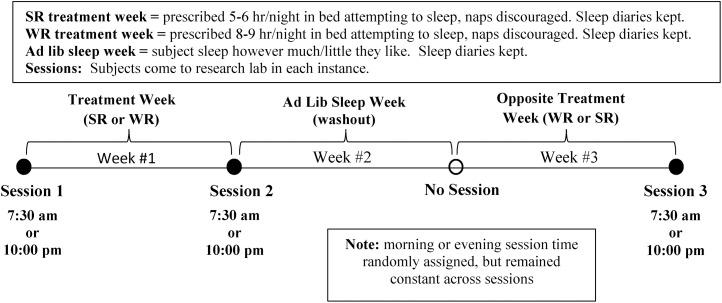
Protocol details and timeline.

The 3-week protocol was approved by the Institutional Review Board (IRB) in the Office of Research Protection at Appalachian State University. Informed written consent was obtained in person from each subject by the experimenter, as approved by the IRB. Fig 1 shows the study time line. Subjects attended 3 lab sessions, with each at the same time of day (determined by random assignment, as per above) and on the same day of the week. We conducted sessions with groups of 4-20 subjects. Session 1 involved Informed Consent, assignment of an actigraphy device and sleep diaries, instructions on their usage, and review of the study time line. The first week immediately following Session 1 was either the WR or SR condition. The final two weeks involved a wash-out week of ad lib sleep followed by the opposite WR/SR condition (see Fig 1). Sessions 2 and 3 occurred in the lab at the end of each WR/SR week and involved assessment of study outcomes. Throughout the 3-week protocol, we sent emails every 2-3 days to maintain subject contact, remind them of the current sleep week prescription, and to send a cautionary note regarding drowsiness and safety during the SR week. Control subjects followed the same procedures as the experimental subjects, except they were prescribed the WR sleep schedule for both weeks 1 and 3, and they were always tested at 11:00 a.m.

### Data analysis

We examined predictors of a subject showing up for Session 1, conditional on having signed up, as well as predictors of finishing the protocol (data can be accessed in the S1 Data file). Both analyses utilized a linear probability estimation model with the following predictors: a) being assigned to SR as the first condition; b) being assigned to morning test sessions; c) age; d) gender; e) self-reported sleep debt; f) PHQ-2 (depression) score; g) GAD-7 (anxiety) score; h) ESS score; i) rMEQ score; j) circadian match/mismatch assignment. We analyzed predictors of compliance in a similar fashion, except that we excluded the variable “being assigned SR first” given that we only included those finishing the protocol for that analysis. Also, “optimal sleep level” was included because those with a high subjective need for sleep might find it even more difficult to comply with SR. For the compliance analysis, one could use “time-in-bed” (i.e., are they in bed 5-6 hours/night for SR and 8-9 hours/night for WR?) or “total sleep time” (TST) as the dependent variable. Because the goal of the sleep manipulation was to restrict sleep quantity during SR relative to WR, we used TST as our dependent variable measure of compliance.

We then examined the impact of the SR protocol on subjective sleepiness (the Karolinska sleepiness scale, or KSS) by estimating a random-effects generalized least-squares model to account for the fact that we have two observations of sleepiness scores on each subject. We estimated two models, each defining SR differently. First, we used actigraphy-measured “average nightly TST” from the week prior to testing, with the assumption that less sleep equates to greater SR. Second, because there is heterogeneity in nightly optimal sleep levels across individuals, we constructed a variable to proxy for one’s personal level of SR during each week (*PersonalSR*). This variable is defined as the subject’s self-reported optimal level of nightly sleep (from the online survey) minus that subject’s actigraphy measured nightly sleep for the week preceding testing. Thus, we calculated a personal SR measure for each subject for each week. In estimating predictors of sleepiness, we also entered demographic, session-level, and depression/anxiety variables, as well as ESS, chronotype, and the circadian mismatch status of the test session.

Partway through our study, we added the PANAS affective rating scale [35] to the subjective sleepiness instrument administered during Sessions 2 and 3 (S1 Appendix). So, for a subset of our sample (n = 80 experimental subjects), we generated data on self-reported affective states that could lend further evidence on protocol performance (S1 Data file). The PANAS instrument generates a self-report on 20 distinct measures of positive and negative affect. For our purposes, we focused on the mood states of “irritability” and “alertness”, and the mood states analyses are parallel to those used in examining self-reported sleepiness.

## Results

### Subjects

In total, 3630 individuals completed the online survey, 256 agreed via email response to participate in the 3-week study, 221 of these showed for the initial in-person session (i.e., 35 no-shows), and 37 subjects dropped out at some point during the protocol, which leaves a total of 184 subjects who completed the protocol. Of those completing the protocol, actigraphy malfunctions caused lost data on 9 subjects, and so we completed the study with 175 total subjects of intact actigraphy data (n = 145 experimental and n = 30 control subjects). Table 2 shows summary statistics from the online sleep survey of those who signed up for our experiment, separated into those who did or did not complete the protocol. Table 3 shows treatment week summary statistics on the sleep levels of the 175 subjects who completed the study *and* had complete actigraphy data. Regarding compliant treatment subjects, actigraphy measured sleep was 48.04 ± 60.16 min less per night than what was self-reported (from online survey) as personally optimal during the WR condition but 154.70 ± 59.44 min less per night than the personal optimal level during SR.

**Table 2:** Summary statistics from initial online survey.

**DID NOT FINISH STUDY**					
***1***^***st***^ ***Session no-shows (n = 35) & attrition (n = 37)***	**Obs**	**Mean**	**Std Dev**	**Min**	**Max**
Age	72	21.54	3.74	18	38
Female (0,1)	72	.57	.50	0	1
Optimal nightly sleep hrs/night (self-report)	72	8.18	1.06	5	10
PHQ-2 (Depression)	72	.88	.82	0	2
GAD-7 (Anxiety)	72	4.44	2.73	0	9
Epworth	72	7.82	3.19	2	14
rMEQ score	72	11.39	5.00	4	21
**FINISHED STUDY**	**Obs**	**Mean**	**Std Dev**	**Min**	**Max**
Age	184	21.37	4.31	17^	39
Female (0,1)	184	.60	.49	0	1
Optimal Sleep (self-report)	184	8.03	.97	4	11
PHQ-2 (Depression)	184	.69	.80	0	2
GAD-7 (Anxiety)	184	4.51	2.37	0	9
Epworth	184	7.97	3.44	1	18
rMEQ score	184	12.19	4.96	4	24

^The required age for participation in the study was 18 years old, and it was verified that this one subject completed the online survey while still 17 but turned 18 years old prior to recruitment for the main study.

**Table 3:** Summary statistics on actigraphy-measured average sleep (min/night).

**Control Subjects** *(weeks 1 & 3 prescribed to WR)*	**Obs**	**Mean**	**Std Dev**	**Min**	**Max**
Week 1 (WR)	30	436.60	32.42	317.43	493.57
Week 2 (ad lib sleep)	30	422.49	47.93	293.43	513.22
Week 3 (WR)	30	436.55	28.60	347.57	478.07
**Treatment Subjects** *(includes non-compliant subjects)*	**Obs**	**Mean**	**Std Dev**	**Min**	**Max**
WR Week (Week 1 or 3)	145	429.88	32.39	337.43	510.50
Week 2 (ad lib sleep)	144^	427.70	43.18	328.64	606.92
SR Week (Week 3 or 1)	145	336.73	37.06	225.28	479.22

^One subject had an actigraphy malfunction only during the ad lib sleep week. Because complete sleep data that week was not necessary for analysis of the protocol validity characteristics, that subject’s data were still included in the analysis.

### Attrition

Table 4 shows results from models predicting whether a subject showed up for Session 1 (Model 1) or finished the protocol, conditional on showing up for Session 1 (Model 2). Model 1 in Table 4 shows that no available demographic or session characteristic predicted the likelihood of showing up for Session 1 (i.e., no-shows are not systematic, but rather somewhat random). Model 2 shows that study completion was more likely for those with lower depression scores, higher anxiety scores, and for those assigned to the morning test sessions. Recall that depression and anxiety scores in these estimations are sub-clinical levels, because our screening criteria excluded those considered at clinical risk for either anxiety or depressive disorder. Given the depression and anxiety findings, we included these measures in our other analyses to remove any effect they may have on outcomes, via their impact on selection into the final sample.

**Table 4:** Determinants of protocol completion.

	Dep Var = *SHOWED UP DAY 1*	Dep Var = *FINISHED PROTOCOL*
	All Recruited Treatment Subjects (n = 256)	Conditioned on Subjects Showing up Day 1 (n = 221)
Variable	Model 1	Model 2
Constant	-.063 (.238)	.894 (.268)***
*SR First* (= 1)	.042 (.044)	-.047 (.051)
*Morning Session* (= 1)	.040 (.045)	.119 (.053)**
Age	-.002 (.005)	-.007 (.006)
Female (= 1)	-.049 (.045)	-.019 (.053)
Optimal Sleep hrs (self-report)	.030 (.022)	-.0004 (.026)
Depression Risk	.010 (.030)	-.059 (.035)*
Anxiety Risk	.012 (.010)	.024 (.011)**
Epworth Sleepiness	-.006 (.007)	-.003 (.008)
rMEQ score	-.003 (.005)	.003 (.005)
*Mismatched* (= 1)	.030 (.045)	-.013 (.052)
R-squared	.0425	.0586

Notes:

*, **, *** indicate significance at the .10, .05, and .01 levels, respectively. Results are similar if estimated using nonlinear Probit estimation technique. Results are also similar if one estimates the models using only the treatment subjects (Model 1, n = 220; Model 2, n = 188). There was a limited number of minority (n = 19) and non-student (n = 18) subjects in our sample. Minority or nonstudent status are both insignificant if included, and their inclusion does not impact the significance of our other variables in any way. There is simply not a large enough subsample on minority or nonstudent status to expect a powerful test of their potential impact in our data.

### Compliance

**Fig 2:**
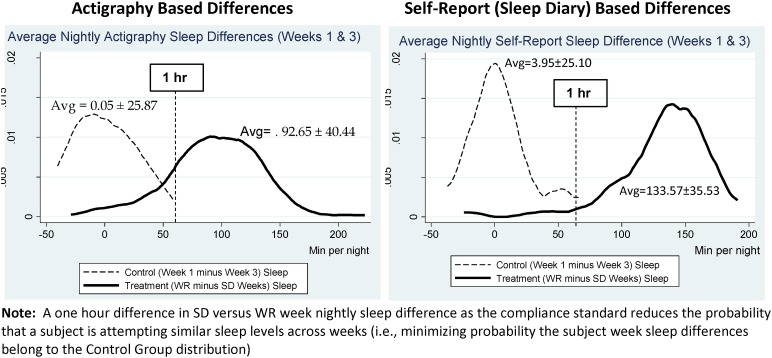
Sleep week difference distributions (control vs. treatment subjects).

Experimental subjects spent an average of 506.9 ± 29.7 min/night time-in-bed during the WR week and an average of 387.5 ± 41.5 min/night time-in-bed during the SR week. Our primary outcome variable for compliance was the extent to which the protocol generated differential TST across the two weeks for a given subject. Fig 2 shows the distribution of within-subject differences in nightly TST between treatment weeks for actigraphy-derived (left) and diary-derived (right) sleep. As expected, control subjects showed no statistical difference in average nightly sleep between weeks #1 and #3. In contrast, experimental subjects slept approximately an hour and a half less each night of the SR week, relative to the WR week, though there is heterogeneity across subjects. The vertical line in Fig 2 represents a 60-minute difference between nightly average sleep in the WR versus SR conditions. Note that this 60-minute difference is a level where a treatment subject looks statistically different from a control subject. Thus, that cutoff point—60 minutes less objective nightly sleep during SR compared to WR—can be considered an empirically-derived compliance standard. By that standard, 82.07% (119/145) of the subjects were compliant. Accordingly, one can define compliance as a continuous variable indicating degree of compliance as shown in Fig 2 (left panel), or compliance can be defined as an empirically derived dichotomous variable. No available demographic or session characteristic predicted compliance, based on either definition (Table 5). Fig 2 also highlights the importance of objective sleep level data in assessing compliance, as the right-panel in Fig 2 shows a clear bias in diary self-reports in direction of the prescribed sleep conditions.

**Table 5:** Determinants of compliance (n = 145 treatment subject observations).

	Model 1: Compliant or Not	Model 2: Degree of Compliance
	Dep Var = *Compliant* = 0,1	Dep Var = *SleepDif*
Variable	Probit estimation	*Ordinary Least Squares Estimation*
Constant	.554 (.368)	54.118 (37.888)
*Morning Session* (= 1)	.035 (.067)	4.933 (6.917)
Age	.003 (.008)	1.057 (.855)
Female (= 1)	-.007 (.069)	-1.00 (7.135)
Optimal Sleep hrs (self-report)	.015 (.035)	.933 (3.655)
Depression	.033 (.047)	.909 (4.824)
Anxiety	-.003 (.016)	-.020 (1.638)
Epworth Sleepiness	.005 (.010)	.374 (1.027)
rMEQ score	.004 (.007)	.568 (.701)
*Mismatched* (= 1)	-.060 (.066)	-6.887 (6.846)
R-squared	0.02	0.04

**Note:** There were no significant predictors in these models. Results are similar for Model 1 if using a nonlinear Probit estimation model.

### Subjective sleepiness and mood states

**Fig 3:**
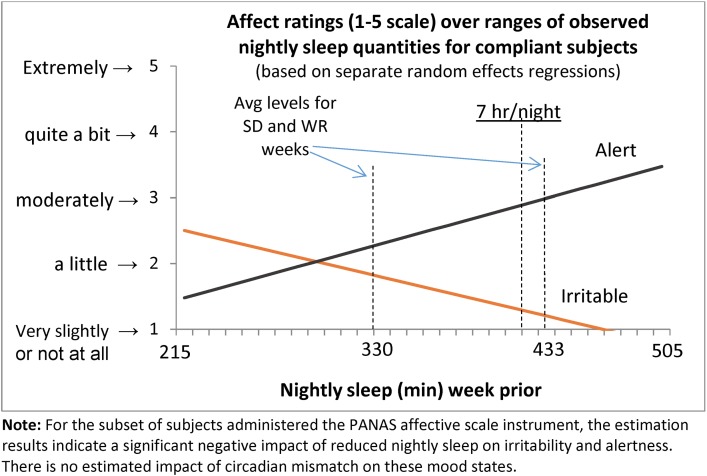
Self-report mood ratings on n = 80 subject subset of data.

The main SR and circadian mismatch results are highlighted in Table 6. Model 1 uses TST the week prior to testing as the independent variable for SR, and Model 2 examines uses the constructed variable *PersonalSR*. After controlling for demographics, ESS, and other session-specific factors, we estimated a statistically significant increase in state sleepiness as measured with the Karolinska Sleepiness Scale (KSS) due to both restricted sleep levels and circadian mismatch. Other consistent findings across models include: morning sessions predict lower KSS scores, lower ESS scores predict lower KSS scores, and male subjects had significantly lower KSS scores. Results of SR and circadian mismatch on mood (i.e., irritability and alertness) are shown in Table 7 and Fig 3. Only SR was estimated to significantly impact self-reported irritability and alertness.

**Table 6:** Determinants of Karolinska sleepiness scale scores.

Variable	Model 1	Model 2
Constant	10.964 (.874)***	2.6714 (.7246)***
*Age*	.034 (.024)	.0473 (.0264)*
*Female* (= 1)	1.195 (.213)***	.8689 (.2305)***
*Session3* (= 1)	-.297 (.199)	-.3776 (.2168)*
*Depression*	-.201 (.139)	-.2795 (.1518)*
*Anxiety*	-.078 (.047)*	-.00418 (.0508)
*Epworth*	.070 (.030)***	.074 (.032)**
*Personal SR*	——	.0105 (.0014)***
*Nightly Sleep Week Prior*	-.017 (.002)***	——
*Morning Session* (= 1)	-.609 (.204)***	-.6573 (.2221)***
rMEQ score	-.019 (.020)	.00887 (.0219)
*Mismatched* (= 1)	.719 (.202)***	.8555 (.2221)***
Wald chi-squared test of model	156.85***	96.43***

Notes

*, **, *** indicate significance at the .10, .05, and .01 levels, respectively. *Session3* = 1 indicates the 2nd lab test session. Key results are robust to defining sleep restriction dichotomously (*SR* = 0,1). In this case, the coefficient on *SR* is positive (2.0608) and statistically significant (p<.01), which is about 3 times the magnitude of the significant (p<.01) and positive coefficient on *Circadian Mismatched* of .6248 in that specification.

**Table 7:** Determinants of irritability and alertness (n = 80).

Variable	*Irritability*	*Alertness*
Constant	3.774 (.699)***	.766 (.676)
Age	.011 (.020)	-.024 (.019)
Female (= 1)	.187 (.172)	-.494 (.167)***
Session3 (= 1)	-.154 (.138)	-.133 (.133)
Depression	-.064 (.109)	.062 (.106)
Anxiety	.043 (.036)	.041 (.035)
*Epworth*	.039 (.027)	-.029 (.026)
*Nightly Sleep Week Prior*	-.006 (.001)***	.007 (.001)***
*Morning Session* (= 1)	-.133 (.165)	.149 (.160)
rMEQ score	-.017 (.017)	.012 (.017)
*Mismatched* (= 1)	-.097 (.167)	-.236 (.162)
Wald chi-squared test of model	35.76***	42.83***

Note:

*** indicates significance at the .01 level. One subject failed to fill out the PANAS during one of the sessions, hence there is only one observation of PANAS ratings for that subject. Results are robust to the use of the *Personal SR* or dichotomous *SR*(= 0,1) variable in place of *Nightly Sleep Week Prior*.

## Discussion

This study aimed to build on prior work where sleep schedules were manipulated outside the laboratory in an effort to study SR in more naturalistic settings [10,11,12,13,14]. We developed a method for implementing an experimental sleep restriction and circadian mismatch protocol outside the sleep-laboratory setting. Our first goal was to enforce a WR and SR sleep schedule in order to create a significant difference in TST between the two treatment weeks. A second goal was to utilize a circadian mismatch protocol to test the impact of circadian mismatch during the biological day. We also assessed whether the two manipulations (SR and circadian) produced the expected changes in subjective sleepiness and mood states. Overall, our protocol was successful at achieving these goals. Subjects obtained, on average, about 1.5 hours less nightly sleep during the SR week compared to the WR week (Fig 2), and our recruitment and randomization procedures allowed us to test approximately equal numbers of self-reported MT and ET subjects during morning and evening test sessions. Both manipulations reliably increased KSS scores, while only the SR manipulation reliably altered self-reported mood states. We note, however, that the SR manipulation was a statistically more powerful repeated-measures design component, while the circadian mismatch component of the study was between-subjects.

To put the KSS (sleepiness) scores data in a broader context, other studies find KSS ≈ 6.0-7.5 following 5 nights of SR [36,37] and KSS ≈ 5.0-6.5 following 1-2 nights of SR [38,39,16] (existing studies often only show such data graphically and do not provide exact means or standard deviations). In comparison, average KSS score among compliant subjects in our data is 6.55 ± 1.57 during SR and 4.45 ± 1.70 during WR, which is in line with the literature. As noted previously, we did not prohibit compensatory behaviors to combat sleepiness in our study. Extra sleep or naps were not endorsed, but to the extent they may have occurred, this would have increased one’s total sleep time and negatively impacted compliance. Daytimes naps in at-home studies are typically discouraged or prohibited [13,16] but, since naps did not *per se* disqualify our subjects, the reader might wonder to what extent subjects in our study took naps or if naps contributed towards noncompliance. In our sample, we identify from the sleep diary and actigraphy records 34 subjects (of 145 treatment subjects) who self-reported at least one nap during a treatment week. Of these 34 “nappers” (some of whom took only one nap the entire treatment week), we identify 9 who were noncompliant. Of these 9 subjects, we use the actigraphy records to document that only 3 of those noncompliant nappers were deemed noncompliant because of their nap(s). In other words, of the relatively few nappers who had noncompliant sleep levels, the majority were deemed noncompliant due to their nightly sleep episode data and not their naps.

**Fig 4:**
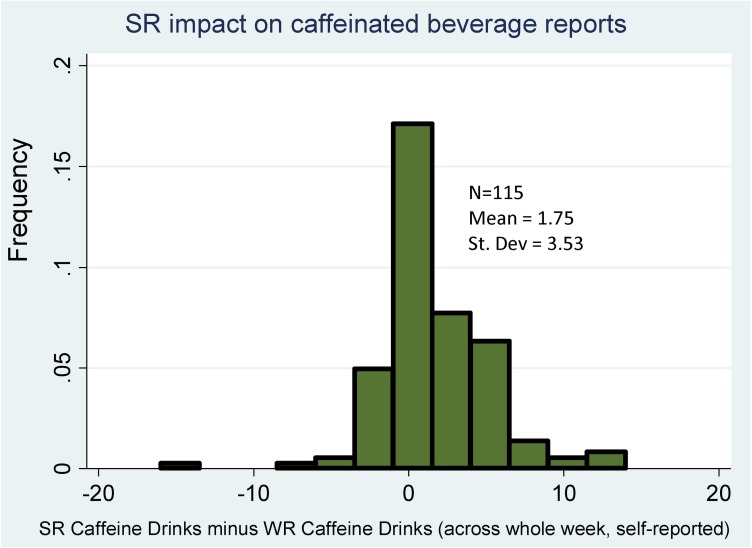
Within-subjects difference in self-reported caffeine use (SR-WR week).

The key compensatory behavior for which we elicited data was subject daily caffeine use, which was self-reported on the subjects’ sleep diaries. The data on daily number of caffeinated beverages were summed for each subject across all days of the treatment week. The average weekly number of caffeinated beverages consumed by compliant treatment subjects (n = 115 complete diary records) was 4.62 ± 3.58 during the SR week and 3.54 ± 3.73 during the WR week. We also constructed the subject-specific difference in number of caffeinated beverages between the SR and WR treatment weeks, the distribution of which is shown in Fig 4. A one-sample t-test confirms significantly more self-reported caffeine use by subjects during SR (t-statistic = 5.30, *p* <.01), which confirms caffeine use as an important compensatory behavior used by subjects. Unfortunately, we did not find comparable mood data in the existing literature, as overall measures or distinct mood instruments using different scoring are sometimes used. However, our result that irritability increased and alertness decreased with SR is consistent with the literature in a general sense.

A key feature of the protocol’s external validity is that it allows subjects to generally go about their lives as usual and in their usual surroundings. This same feature, though, raises concerns about both early attrition (will subjects show up for each in-lab test session?) and completion (will they finish the study?). Since recruited subjects who fail to show up for Session 1 impose a cost on the investigator, it would be useful to know what predicts showing up for Session 1. Of course, it is even more costly for the investigator if someone drops-out of the study, and so the predictors of study completion are even more valuable. Unfortunately, none of the demographic or session-level variables we measured were able to predict attrition prior to Session 1. However, we did identify variables predicting completion of the protocol conditional on the subject having showed up for Session 1. While subjects scoring higher on the PHQ-2 (depression screener) were less likely to finish the protocol, subjects scoring higher on the GAD-7 (anxiety screener) were actually more likely to finish the protocol, holding all else constant. We found these results despite excluding those screened as high risk for depression or anxiety. In other words, even subclinical levels of mental health symptoms may affect study completion. We also found that subjects were significantly more likely to finish the 3-week protocol if randomly assigned to a Morning Session. One might speculate that subjects who voluntarily signed up for an early morning commitment may be more likely to stick to that commitment, or perhaps there is simply a higher likelihood that additional distractions can build up during the day and thereby decrease the likelihood of attending the Evening session.

One critical question in evaluating the viability of this protocol is whether subjects complied with the sleep schedule, given the other demands of life outside the laboratory. Our data suggest overall good compliance, with expected variability. The prescribed sleep schedules allowed for a minimum of 2 hours difference in nightly time-in-bed between the two weeks (i.e., SR allowed a maximum of 6 hours in bed, WR allowed a minimum of 8 hours). Actigraphy-based data showed our subjects spent almost exactly 2 hours less in bed and obtained an average of 1.5 hours less sleep each night during SR, relative to WR, with 82% of subjects obtaining at least 60 minutes less average nightly sleep during SR. In this context, the two panels in Fig 2 highlight the importance of objective actigraphy-based data in naturalistic experiment settings. Specifically, it is clear from the right-panel of Fig 2. that subjects’ self-reported sleep levels (from diaries) seem to be biased in favor of the experimental sleep prescription. Control subjects’ week 1 versus week 3 nightly sleep differences have a more pronounced spike at a difference of zero, and the self-reported difference in nightly sleep during WR compared to SR is over 40 minutes larger in the diary data compared to actigraphy. It is also noteworthy that none of the session-level variables predicted compliance, suggesting there were no particular aspects of the protocol that created difficulty in compliance for the participants. It is important that a protocol designed for use outside the lab does not have built-in barriers to compliance.

We found evidence for the validity of the protocol in its ability to manipulate sleepiness and mood states of the subjects. As expected, SR and circadian misalignment both increased subjective levels of sleepiness. Given that we were able to reliably increase sleepiness, this suggests that these manipulations can be used to examine other measures, such as cognition, which is typically studied with more resource intensive in-lab procedures. Thus, we believe this protocol provides an alternative methodology to examine the impact of sleep loss and circadian misalignment when an investigator wants to actively manipulate those constructs and yet still allow individuals to live in their natural environments.

We end with a precautionary note to investigators considering future at-home SR protocols. It is well known that SR over the course of several days impairs sustained attention, increases reactions times, and can produce micro-sleeps [1,4,15,28]. A myriad of observational studies has also documented increased accident risk with SR [25,26,27]. It is incumbent upon investigators to make potential study participants aware of these risks and to put risk management measures in place, and other researchers may choose to implement screening criteria beyond what we implemented. The exact measures will depend on the specifics of the SR imposed, as well as local regulatory requirements. For example, we detailed risks during the informed consent procedures, and included the following cautionary message in the email reminders sent to subjects every 2-3 days during the study:

“Because part of this study involves a sleep restriction condition, please remember to be cautious regarding the activities you take part in during the chronic sleep restriction condition of this study and/or whenever you are operating at times of day where you might normally be sleeping. In particular, driving or operating dangerous machinery or doing other activities when drowsy may put you and others at increased risk of serious or even fatal accident or injury. Please keep this in mind as you consider making alternative arrangements for transportation or activities you undertake while part of this study.”

Additionally, we allowed some flexibility in the level of compliance among subjects and, as always, subjects were free to withdraw at any point. Given the nature of the study, which was to test a protocol that would allow larger-scale data collection without the intensive oversight of subjects required in a laboratory-based study, we did not contact those who failed to arrive for one of the sessions in order to determine a reason for drop-out. Thus, we cannot rule out the possibility that some subjects dropped out due to struggling with SR (or, if not showing up for Session 1, anticipating struggling with SR). However, our data show that rates of attrition, whether for those failing to show up on Session 1 or for those who later dropped out, were not significantly different between those assigned SR during week #1 compared to WR in week #1 (2-sample proportions test, *p* > .10, in both instances).

Other researchers may also wish to gather more detailed data on subject experiences during treatment condition weeks, or implement precautionary measures in addition to ours. For example, a limitation in our study is that we did not explicitly caution subjects against drinking alcohol during the SR condition, though alcohol consumption may worsen sleepiness related symptoms [40,41,42]. We suggest that other researchers caution subjects against the use of alcohol during SR, and we will include such a warning in future studies as an additional risk management measure. Recall that subjects could engage in whatever compensatory behaviours they wanted, apart from sleeping. While this somewhat reduces experimental control, such allowances (e.g., caffeine use) further added to the ecological validity of the protocol and constituted a risk management measure that likely helped prevent adverse outcomes in this study. Finally, we note that some subjects extended sleep during the SR week or engaged in occasional naps presumably to combat sleepiness. Such behaviors entered into the scored sleep data and the assessment of subject compliance. Future studies may require, and/or investigators may desire, different measures to assess compensatory behaviours more systematically.

## Supporting information

S1 Appendix Prescreen and test session survey instruments. (DOCX)

S1 Data Raw data on all subjects. (XLSX)

## References

[1] Dinges DF, Pack F, Williams K, Gillen KA, Powell JW, Ott GE, et al Cumulative sleepiness, mood disturbance, and psychomotor vigilance performance decrements during a week of sleep restricted to 4-5 hours per night. Sleep. 1997; 20(4):267 9231952

[2] Cajochen C, Khalsa SB, Wyatt JK, Czeisler CA, Dijk DJ. EEG and ocular correlates of circadian melatonin phase and human performance decrements during sleep loss. Am J Physiol. 1999; 277(3 Pt 2):R640–49. 10484479 10.1152/ajpregu.1999.277.3.r640

[3] Wright KP Jr, Hull JT, Czeisler CA. Relationship between alertness, performance, and body temperature in humans. Am J Physiol-Reg I. 2002; 283(6):R1370–77. 10.1152/ajpregu.00205.2002 12388468

[4] Van Dongen HP, Maislin G, Mullington JM, Dinges DF. The cumulative cost of additional wakefulness: dose-response effects on neurobehavioral functions and sleep physiology from chronic sleep restriction and total sleep deprivation. Sleep. 2003; 26(2):117–26. 12683469 10.1093/sleep/26.2.117

[5] Drummond SP, Anderson DE, Straus LD, Vogel EK, Perez VB. The effects of two types of sleep deprivation on visual working memory capacity and filtering efficiency. PLoS One. 2012; 7(4):e35653 10.1371/journal.pone.0035653 22530064 PMC3329471

[6] Ftouni S, Sletten TL, Howard M, Anderson C, Lenne MG, Lockley SW, et al Objective and subjective measures of sleepiness, and their associations with on-road driving events in shift workers. J Sleep Res. 2013; 22(1):58–69. 10.1111/j.1365-2869.2012.01038.x 22861524

[7] Rajaratnam SM, Howard ME, Grunstein RR. Sleep loss and circadian disruption in shift work: health burden and management. Med J Aust. 2013; 199(8):S11–15. 24138359 10.5694/mja13.10561

[8] Marquie JC, Tucker P, Folkard S, Gentil C, Ansiau D. Chronic effects of shift work on cognition: findings from the VISAT longitudinal study. Occup Environ Med. 2015; 72(4):258–64. 10.1136/oemed-2013-101993 25367246

[9] Magee M, Sletten TL, Ferguson SA, Grunstein RR, Anderson C, Kennaway DJ, et al Associations between number of consecutive night shifts and impairment of neurobehavioral performance during a subsequent simulated night shift. Scand J Work Environ Health. 2016; 42(3):217–27. 10.5271/sjweh.3560 27064758

[10] Fallone G, Seifer R, Acebo C, Carskadon MA. How well do school-aged children comply with imposed sleep schedules at home? Sleep. 2002; 25(7):739–45. 12405609 10.1093/sleep/25.7.739

[11] Sadeh A, Gruber R, Raviv A. The Effects of Sleep Restriction and Extension on School‐Age Children: What a Difference an Hour Makes. Child Dev. 2003; 74(2):444–55. 12705565 10.1111/1467-8624.7402008

[12] Fallone G, Acebo C, Seifer R, Carskadon MA. Experimental restriction of sleep opportunity in children: effects on teacher ratings. Sleep. 2005; 28(12):1561–67. 16408416 10.1093/sleep/28.12.1561

[13] Beebe DW, Fallone G, Godiwala N, Flanigan M, Martin D, Schaffner L, et al Feasibility and behavioral effects of an at‐home multi‐night sleep restriction protocol for adolescents. J Child Psychol Psyc. 2008; 49(9):915–23. 10.1111/j.1469-7610.2008.01885.x 18564072

[14] Sadeh A, Dan O, Bar-Haim Y. Online assessment of sustained attention following sleep restriction. Sleep Med. 2011; 12(3):257–61. 10.1016/j.sleep.2010.02.001 20382072

[15] Van Dongen HPA, Baynard MD, Maislin G, Dinges DF. Systematic interindividual differences in neurobehavioral impairment from sleep loss: Evidence of trait-like differential vulnerability. Sleep. 2004; 27(3):423–33. 15164894

[16] Sletten TL, Segal AY. Flynn-Evans EE, Lockley SW, Rajaratnam SM. Inter-Individual Differences in Neurobehavioural Impairment following Sleep Restriction Are Associated with Circadian Rhythm Phase. PloS One. 2015; 10(6): e0128273 10.1371/journal.pone.0128273 26043207 PMC4456409

[17] Sadeh A, Acebo C. The role of actigraphy in sleep medicine. Sleep Med Rev. 2002; 6:113–24. 12531147 10.1053/smrv.2001.0182

[18] Dickinson DL, Drummond SPA, Dyche J. Voluntary sleep choice and its effects on Bayesian decisions. Behav Sleep Med, 2016; 14(5):501–13. 10.1080/15402002.2015.1028064 26507556

[19] Dickinson DL, McElroy T. Circadian effects on strategic reasoning. Exp Econ. 2012; 15(3):444–59.

[20] Schoenborn CA, Adams PF. Health behaviors of adults: United States, 2005-2007. National Center for Health Statistics. *Vital Health Statistics*, *series 10*, *Data from the National Health Survey*, 2010; 245:1–132. 20669609

[21] Bodenhausen GV. Stereotypes as judgmental heuristics: Evidence of circadian variations in discrimination. Psychol Sci. 1990; 1(5):319–22.

[22] Kruglanski AW, Pierro A. Night and Day, You Are the One On Circadian Mismatches and the Transference Effect in Social Perception. Psychol Sci. 2008;19(3):296–301. 10.1111/j.1467-9280.2008.02083.x 18315804

[23] Chelminski I, Petros TV, Plaud JJ, Ferraro FR. Psychometric properties of the reduced Horne and Östberg questionnaire. Pers Indiv Differ. 2000; 29(3):469–78.

[24] Castillo M, Dickinson DL, Petrie R. Sleepiness, choice consistency, and risk preferences. Theory 12 2016, *in press*.

[25] Van Dongen HPA, Kerkhof GA. Predicting cognitive impairment and accident risk. *Human Sleep and Cognition*, *Part II*: *Clinical and Applied Research*. 2011; 190:155. 10.1016/B978-0-444-53817-8.00010-4 21531251

[26] Hershner SD, Chervin RD. Causes and consequences of sleepiness among college students. Nat Sci Sleep. 2014; 6:73–84. 10.2147/NSS.S62907 25018659 PMC4075951

[27] Anderson C, Grunstein RR, Rajaratnam SMW. Hours of work and rest in the rail industry." Intern Med J. 2013; 43(6):717–721. 10.1111/imj.12159 23745994

[28] Belenky G, Wesensten NJ, Thorne DR, Thomas ML, Sing HC, Redmond DP, Russo MB, Balkin TJ. Patterns of performance degradation and restoration during sleep restriction and subsequent recovery: A sleep dose‐response study. J. Sleep Res. 2003; 12(1):1–12. 12603781 10.1046/j.1365-2869.2003.00337.x

[29] Spitzer RL, Kroenke K, Williams JB, Löwe B. A brief measure for assessing generalized anxiety disorder: the GAD-7. Arch Int Med. 2006; 166(10):1092–1097. 16717171 10.1001/archinte.166.10.1092

[30] Kroenke K, Spitzer RL, Williams JB. The Patient Health Questionnaire-2: validity of a two-item depression screener.” Med Care. 2003; 41(11):1284–1292. 10.1097/01.MLR.0000093487.78664.3C 14583691

[31] Adan A, Almirall H. Horne & Östberg morningness-eveningness questionnaire: A reduced scale. Pers Indiv Differ. 1991; 12(3):241–53.

[32] Horne JA, Östberg O. A self-assessment questionnaire to determine morningness-eveningness in human circadian rhythms. Int J Chronobiol. 1976; 4(2):97–110. 1027738

[33] Johns MW. A new method for measuring daytime sleepiness: the Epworth sleepiness scale. Sleep. 1991; 14(6):540–5. 1798888 10.1093/sleep/14.6.540

[34] Sadeh A. The role and validity of actigraphy in sleep medicine: An update. Sleep Med Rev. 2011; 15(4):254–67 10.1016/j.smrv.2010.10.001 21237680

[35] Watson D, Clark LA, Tellegen A. Development and validation of brief measures of positive and negative affect: The PANAS scales. J Pers Soc Psychol. 1988; 54(6):1063 3397865 10.1037//0022-3514.54.6.1063

[36] Åkerstedt T, Kecklund G, Axelsson J. Effects of context on sleepiness self‐ratings during repeated partial sleep deprivation. Chronobiol Int. 2008; 25(2-3):271–278. 18484365 10.1080/07420520802110589

[37] Banks S, Van Dongen HPA, Maislin G, Dinges DF. Neurobehavioral dynamics following chronic sleep restriction: dose-response effects of one night for recovery. Sleep. 2010; 33(8):1013–1026. 20815182 10.1093/sleep/33.8.1013 PMC2910531

[38] Anderson C, Horne JA. A high sugar content, low caffeine drink does not alleviate sleepiness but may worsen it. Hum Psychopharm Clin. 2006; 21(5):299–303. 10.1002/hup.769 16856218

[39] Anderson C, Horne JA. Placebo response to caffeine improves reaction time performance in sleepy people. Hum Psychopharm Clin. 2008; 23(4):333–336. 10.1002/hup.931 18350573

[40] Lee J, Manousakis J, Fielding J, Anderson C. Alcohol and sleep restriction combined reduces vigilant attention, whereas sleep restriction alone enhances distractibility. Sleep. 2015; 38(5):765–775. 10.5665/sleep.4672 25515101 PMC4402669

[41] Barrett PR, Horne JA, Reyner LA. Early evening low alcohol intake also worsens sleepiness‐related driving impairment. Hum Psychopharm Clin. 2005; 20(4):287–290. 10.1002/hup.691 15912483

[42] Vakulin A, Baulk SD, Catcheside PG, Anderson R, van den Heuvel CJ, Banks S, McEvoy RD. Effects of moderate sleep deprivation and low-dose alcohol on driving simulator performance and perception in young men. Sleep. 2007; 30(10):1327–33. 17969466 10.1093/sleep/30.10.1327 PMC2266279

