# Supplementary material for: The viability of an ecologically valid chronic sleep restriction and circadian timing protocol: An examination of sample attrition, compliance, and effectiveness at impacting sleepiness and mood
Source: PLoS One. 2017 Mar 20;12(3):e0174367. doi: 10.1371/journal.pone.0174367 (PMC5358873; doi:10.1371/journal.pone.0174367)
Supplement: S1 Appendix — (DOCX) [file pone.0174367.s001.docx]

**Supporting Information Appendix:**

**Online Pre-screen Survey Questions (answered prior to recruitment)**

(some responses elicited using slider bars, matrix response boxes, or other standard online survey features. For space considerations, questions below are not separated for page breaks as they were in online survey (e.g, to separate basic demographics from rMEQ question block and other questions)

What is your gender?

- Female
- Male

Please enter your valid email address (e.g., [johndoe@emailserver.com](mailto:johndoe@emailserver.com))

This is required if you wish to be entered into the database for eligibility and recruitment consideration for future cash-compensation research experiments

What is your ethnicity?

- Hispanic or Latino
- Not Hispanic or Latino

What is your racial category?

- American Indian/Alaska Native
- Asian
- Native Hawaiian or Other Pacific Islander
- Black or African American
- White (Caucasian)
- Mixed
- Other (please specify in text box)

What is your age?

Are you a student, faculty, or staff?

Over the last 2 weeks, how often have you been bothered by the following problems?

(Options for each are “Not at all”, “Several Days”, “Over Half of the Days”, or “Nearly Every Day”)

- Feeling nervous, anxious or on edge
- Not being able to stop or control worrying
- Worrying too much about different things
- Little interest or pleasure in doing things
- Trouble relaxing
- Being so restless that it is hard to sit still
- Becoming easily annoyed or irritable
- Feeling down, depressed, or hopeless
- Felling afraid as if something awful might happen

Considering only your own “feeling best” rhythm, at what time would you get up if you were entirely free to plan your day?

- between 5:00 am and 6:30 am
- between 6:31 am and 8:00 am
- between 8:01 am and 9:30 am
- between 9:31 am and 11:00 am
- between 11:01 am and noon

During the first half-hour after waking up in the morning, how tired do you feel?

- Very tired
- Fairly tired
- Fairly refreshed
- Very refreshed

At what time in the evening do you feel tired and, as a result, in need of sleep?

- Between 8:00 pm and 9:00 pm
- Between 9:01 pm and 10:30 pm
- Between 10:31 pm and 12:30 am
- Between 12:31 am and 2:00 am
- Between 2:01 am and 3:00 am

At what time of the day do you think you reach your “feeling best” peak?

- Between midnight and 4:30 am
- Between 4:31 am and 7:30 am
- Between 7:31 am and 9:30 am
- Between 9:31 am and 4:30 pm
- Between 4:31 pm and 9:30 pm
- Between 9:31 pm and midnight

One hears about “morning” and “evening” types of people. Which ONE of these types do you consider yourself to be?

- Definitely a ‘morning-type’
- More likely a ‘morning-type’ than an ‘evening-type’
- More likely an ‘evening-type’ than a ‘morning-type’
- Definitely an ‘evening-type’

**Over the last 7 nights,** what is the average amount of sleep you obtained each night?

**Last night**, how much sleep did you get?

**What do you feel is the optimal amount of sleep for you personally to get each night?** (optimal in terms of next day alertness, performance, and functionality for you personally.)

How likely are you to doze off or fall asleep in the following situations, in contrast to just feeling tired? This refers to your usual way of life in recent times. Even if you have not done some of these things recently, try to work out how they would have affected you.

(options are “would NEVER doze or fall asleep”, “SLIGHT chance of dozing or falling asleep”, “MODERATE chance of dozing or falling asleep”, or “HIGH chance or dozing or falling asleep”)

- Sitting and reading
- Watching TV
- Sitting, inactive in a public place (e.g., a theater or a meeting)
- As a passenger in a car for an hour
- Lying down to rest in the afternoon when circumstances permit
- Sitting and talking to someone
- Sitting quietly after lunch without alcohol
- In a car, while stopped for a few minutes in traffic

Do you have a diagnosed sleep disorder?

- Yes
- No
- Not diagnosed, but I believe I may have a sleep disorder

If you responded “Yes” (i.e., you have a diagnosed sleep disorder), what is it?

The following was administered during each test session (both at the beginning and end of the session)

**Karolinska Sleepiness Scale**

Please mark the number that best corresponds to how sleepy you feel **right now**. You may mark any number, but mark only one number.

_____ 1. Extremely alert

_____ 2.

_____ 3. Alert

_____ 4.

_____ 5. Neither alert nor sleepy

_____ 6.

_____ 7. Sleepy – but no difficulty remaining awake

_____ 8.

_____ 9. Extremely sleepy – fighting sleep

For the final subset of 80 subjects (as described in main text), we also administered following PANAS mood ratings instrument at the beginning and end of each test session.

**PANAS (Positive and Negative Affect Scale)**

The following scale consists of a number of words that describe different feelings and emotions. Read each item then mark the appropriate answer in the space next to the word. Indicate to what extent you feel this way right now, that is, at the present moment. Use the following scale to record your feelings.

1 2 3 4 5

Very slightly a little moderately quite a bit extremely

Or not at all

| ____interested | ____strong | ____enthusiastic | ____ashamed | ____attentive |
| --- | --- | --- | --- | --- |
| ____distressed | ____guilty | ____proud | ____inspired | ____jittery |
| ____excited | ____scared | ____irritable | ____nervous | ____active |
| ____upset | ____hostile | ____alert | ____determined | ____afraid |
